# Body mass index and risk of subtypes of head-neck cancer: the Netherlands Cohort Study

**DOI:** 10.1038/srep17744

**Published:** 2015-12-04

**Authors:** Denise H. E. Maasland, Piet A. van den Brandt, Bernd Kremer, Leo J. Schouten

**Affiliations:** 1 Department of Epidemiology, GROW - School for Oncology & Developmental Biology, Maastricht University, Maastricht, The Netherlands; 2 Department of Otorhinolaryngology, Head & Neck Surgery, GROW - School for Oncology & Developmental Biology, Maastricht University Medical Center, Maastricht, The Netherlands

## Abstract

Low body mass index (BMI) has been associated with risk of head-neck cancer (HNC), but prospective data are scarce. We investigated the association between BMI, BMI at age 20 years and change in BMI during adulthood with risk of HNC and HNC subtypes. 120,852 participants completed a questionnaire on diet and other cancer risk factors, including anthropometric measurements, at baseline in 1986. After 20.3 years of follow-up, 411 HNC (127 oral cavity cancer (OCC), 84 oro-/hypopharyngeal cancer (OHPC), and 197 laryngeal cancer (LC)) cases and 3,980 subcohort members were available for case-cohort analysis using Cox proportional hazards models. BMI at baseline was inversely associated with risk of HNC overall, with a multivariate rate ratio of 3.31 (95% CI 1.40–7.82) for subjects with a BMI < 18.5 kg/m^2^, compared to participants with a BMI of 18.5 to 25 kg/m^2^. Among HNC subtypes, this association was strongest for OCC and OHPC. The association between BMI at age 20 and HNC risk appeared to be positive. In this large prospective cohort study, we found an inverse association between BMI at baseline and HNC risk. For BMI at age 20, however, a positive rather than inverse association was found.

Worldwide and in Europe, head and neck cancer (HNC) is the seventh most common type of cancer, including malignancies in the oral cavity, pharynx and larynx^1^,^2^. Established risk factors for HNC are cigarette smoking, alcohol consumption and human papillomavirus (HPV) infection^3^,^4^. A low body mass index (BMI) has also been associated with HNC risk, but this association remains to be clarified. In 2007, the World Cancer Research Fund (WCRF) concluded that data regarding the association between body fatness and HNC risk were insufficient to allow any conclusions to be drawn^3^. Several case-control studies investigated the association between BMI and HNC and mostly found inverse associations. However, since case-control studies are prone to bias, it remains unclear whether the results represent a true inverse association between BMI and HNC or an association due to reverse causality, confounding or effect modification^3^,^5^,^6^,^7^. Recently, three prospective cohort studies investigated the association between BMI and HNC risk. In the Prostate, Lung, Colorectal, and Ovarian (PLCO) cohort^8^, BMI at different time points in life was not associated with HNC risk. The Cancer Prevention Study-II (CPS-II) cohort showed no association between BMI and HNC incidence either, although BMI was inversely associated with HNC mortality in smokers^9^. The National Institutes of Health-AARP (NIH-AARP) Diet and Health Study^10^ found that HNC risk was inversely associated with leanness among current smokers, and concluded that the association between leanness and HNC risk may be due to effect modification by smoking.

Given the current evidence, it remains critical to study the association between BMI and HNC risk in prospective cohort studies with comprehensive adjustment for smoking. We therefore examined the association with BMI for HNC and the most frequent HNC subtypes^11^ –i.e., oral cavity cancer (OCC), oro-/hypopharyngeal cancer (OHPC), and laryngeal cancer (LC) – within the large prospective Netherlands Cohort Study (NLCS). In addition to BMI at study baseline, we also studied the effects of BMI at age 20 years and change in BMI during adulthood on HNC risk. Finally, we investigated the association of BMI with HNC risk according to smoking status and alcohol consumption.

## Results

The mean BMI at baseline of subcohort members (25.0 kg/m^2^) and cases (24.8 kg/m^2^) was slightly lower in HNC cases (Table 1). There was a minor difference between subcohort members and cases regarding BMI at age 20 years (21.5 and 21.9 kg/m^2^, respectively), as well as with respect to change in BMI since age 20 years (plus 3.5 and 3.0 kg/m^2^, respectively). Among subcohort members and cases, men generally had a similar mean BMI, whereas female cases had a considerably lower BMI at baseline than both male cases and female subcohort members. Notable characteristics with regard to cigarette smoking and alcohol consumption have been described previously^12^.

To examine possible reverse causation, we evaluated BMI at baseline of HNC cases during the follow-up period. As we expected, there was no clear pattern in BMI at baseline among HNC cases diagnosed in the course of 20.3 years of follow-up (Table 2). HNC overall cases diagnosed after the second year of follow-up (*N* = 378) had a mean BMI at baseline of 24.8 kg/m^2^, whereas cases diagnosed during the first two years of follow-up (*N* = 33) had a mean BMI of 24.9 kg/m^2^, a non-statistically significant difference.

Results from age- and sex-adjusted and multivariable-adjusted analyses showed mostly inverse associations between BMI at baseline and risk of HNC overall and HNC subtypes, although these associations were generally somewhat weaker in multivariable-adjusted analyses (Table 3).

BMI at baseline was inversely associated with risk of HNC overall, with a multivariate rate ratio (RR) of 3.31 (95% confidence interval (CI) 1.40–7.82) for subjects with a BMI < 18.5 kg/m^2^, whereas participants with a BMI ≥ 30 kg/m^2^ had a RR of 0.48 (95% CI 0.22–1.03), both compared to participants with a BMI of 18.5 to 25 kg/m^2^ (Table 3). The association between BMI at baseline and risk of HNC overall was comparable for men and women and no statistically significant interaction with sex was found (*P* = 0.29) for BMI on a continuous scale. Sensitivity analyses showed essentially similar results after exclusion of the first two years of follow-up. We investigated whether the subgroup with underweight at baseline had specific characteristics with regard to smoking and alcohol consumption, since this might have biased the results, but this group was very heterogeneous with regard to these lifestyle aspects.

Among HNC subtypes, BMI at baseline was in general inversely associated with HNC risk as well, with statistically significant associations in OCC (multivariate RR comparing participants with a BMI < 18.5 kg/m^2^ to those with a BMI of 18.5 to 25: 4.49, 95% CI 1.45–13.93) and OHPC (RR: 4.96, 95% CI 1.34–18.33) but not LC (RR: 1.25, 95% CI 0.15–10.31) (Table 3). For LC, however, a statistically significant interaction with sex (*P* = 0.01) was found, with a decreased risk of LC per kg/m^2^ increase in BMI in women (RR: 0.83, 95% CI 0.71–0.97), but the number of female cases was small (*N* = 14). We performed sensitivity analyses with only men (*N* = 183) in categories of BMI at baseline because of this interaction, but these results showed the same pattern as the results for men and women combined (data not shown).

In contrast to the association between BMI at baseline and HNC risk, the association between BMI at age 20 and HNC risk appeared to be positive rather than inverse, with statistically significant associations on the continuous scale (Table 4). Furthermore, point estimates regarding the association between change in BMI since the age of 20 years and HNC risk mostly indicated an inverse association. In addition, we found an interaction between sex and BMI at age 20 years for HNC overall. RRs regarding BMI at age 20 years appeared slightly stronger in multivariable-adjusted analyses compared with age- and sex-adjusted analyses, whereas associations between change in BMI and HNC risk showed both stronger and weaker RRs in multivariate analyses (Table 4).

No statistically significant interaction was found between BMI at baseline and cigarette smoking (*P* for interaction = 0.86) for HNC overall, nor for BMI at age 20 or change in BMI and cigarette smoking (Table 5). A statistically significant interaction was found for both BMI at baseline and at age 20 and alcohol consumption; stratified analyses showed a consistent pattern of the lowest relative risks of HNC overall for BMI at baseline, BMI at age 20, and change in BMI in non-drinkers.

## Discussion

In this large prospective cohort study, we found an inverse association between BMI at baseline and risk of HNC overall. Among HNC subtypes, BMI at baseline showed the strongest inverse association with OCC and OHPC. For BMI at age 20, on the other hand, we found a positive rather than inverse association, whereas the association between change in BMI since the age of 20 years and HNC risk appeared to be inverse again. Finally, there was effect modification by alcohol consumption in our study, with the lowest risks of HNC overall for BMI at baseline, BMI at age 20, and change in BMI in non-drinkers.

Previous studies showed mixed results regarding BMI and HNC risk. Case-control studies largely indicated an inverse association between BMI and HNC risk^3^, but a systematic literature review by the WCRF^3^ concluded that data regarding the association between body fatness and HNC risk —based on case-control studies— were insufficient to allow conclusions to be drawn. Since then, a large pooled analysis^5^ of 17 case-control studies with 12,716 cases and 17,438 controls showed that leanness (BMI < 18.5 kg/m^2^) was associated with increased HNC risk, regardless of smoking and drinking status. Furthermore, three prospective cohort studies examined the association between BMI and HNC risk. The CPS-II cohort^9^ included 340 HNC cases and showed no association between BMI and HNC incidence. There was no effect-modification by smoking status. In the PLCO cohort^8^, with 177 cases, neither BMI at different time points in life nor changes in BMI were associated with HNC risk. Recently, the NIH-AARP Diet and Health Study^10^, which comprised 779 cases, showed evidence for an inverse relationship between BMI at baseline and HNC risk, in particular OCC and OHPC, but none of the associations were statistically significant. In addition, BMI at earlier ages showed no association with HNC risk. When stratified by smoking, the inverse association was only observed among current (and not former) smokers (Hazard Ratio (HR) 0.76 per 5 kg/m^2^ increase, 95% CI 0.63–0.93); also, the association diminished as initial years of follow-up were excluded. None of the three cohort studies investigated effect-modification by alcohol intake.

The results from our prospective cohort study partly confirm findings from previous —both case-control and prospective— studies. As most case-control studies and the NIH-AARP cohort study^10^, we also found an inverse association between BMI and HNC risk, and —like NIH-AARP— with strongest associations for OCC and OHPC. The CPS-II^9^ and the PLCO^8^ cohort, on the other hand, did not find an inverse association between BMI and HNC risk. Unlike previous cohort studies^8^,^10^, we also found a positive association with regard to BMI at age 20 and HNC risk, and an interaction with alcohol consumption. Finally, we did not find an interaction with smoking status, although this might have to do with a lack of power (see below).

The question remains whether the inverse association we found between BMI at baseline and HNC risk is a true effect by BMI, or an effect based on reverse causality or confounding by smoking, alcohol consumption, or other factors. We cannot clearly explain why we found a positive rather than inverse association between BMI at age 20 and HNC risk, whilst BMI at baseline was in general inversely associated with HNC risk. Given the contrast in our results regarding the associations between BMI at baseline, BMI at age 20, and HNC risk, it appears that leanness itself is probably not a causal factor in this association. The fact that some associations were weaker —like NIH-AARP— but others stronger —like NIH-AARP— in multivariable-adjusted analyses than in age- and sex-adjusted analyses implies the possibility of residual confounding. Reverse causality might play a role in the association between BMI at baseline and HNC risk. However, sensitivity analyses showed similar results for different periods of follow-up, which makes reverse causality unlikely.

Strengths of our study are the prospective nature, our large case-number, and the completeness and duration of follow-up. In addition, we had the ability to study HNC subtypes and to adjust for confounders thoroughly. A possible limitation of our study is that the data on BMI in our study are self-reported, which may have led to bias due to misclassification of exposure. BMI at age 20 years was calculated using self-reported weight at age 20 years and this might have introduced recall bias; however, we expect this to be non-differential. Despite thorough adjustment for confounding by smoking and alcohol consumption, we cannot rule out residual confounding, as described above. Furthermore, in stratified analyses, we did not find a statistically significant interaction with regard to cigarette smoking. However, the analysis included only 55 cases among never smokers, mainly females, and there may have been a lack of power to detect a significant interaction. Finally, we lack data on HPV infection^12^.

In conclusion, we found an inverse association between BMI at baseline and HNC risk in this large cohort study. Among HNC subtypes, this association was strongest for OCC and OHPC. For BMI at age 20, however, a positive rather than inverse association was found. Furthermore, associations of BMI with HNC risk may be modified by alcohol consumption. We conclude that leanness itself is probably not a causal factor in the association with HNC. Future studies are warranted for further clarifications of the possible mechanisms involved regarding BMI and HNC risk.

## Methods

### Study design and population

The NLCS was initiated in September 1986 and includes 120,852 participants, aged 55–69 years at baseline^13^. The NLCS has been approved by the institutional review board of the TNO Quality of Life Research Institute (Zeist, the Netherlands) and the Medical Ethics Committee of Maastricht University (Maastricht, The Netherlands). All methods were carried out in accordance with the approved guidelines. All cohort members consented to participate in the study by completing and returning the self-administered questionnaire.

We used the case-cohort design for efficiency in data processing and follow-up^14^. Cases were identified from the entire cohort, whereas the number of person-years at risk for the entire cohort was estimated using a subcohort of 5,000 people who were randomly sampled from the total cohort at baseline. Follow-up for cancer incidence was done by record linkage to the Netherlands Cancer Registry (NCR) and the nationwide network and pathology registry (PALGA)^15^. Follow-up for vital status of the subcohort was nearly 100% complete after 20.3 years and the completeness of cancer follow-up is estimated to be ≥96%^16^.

**Figure 1:**
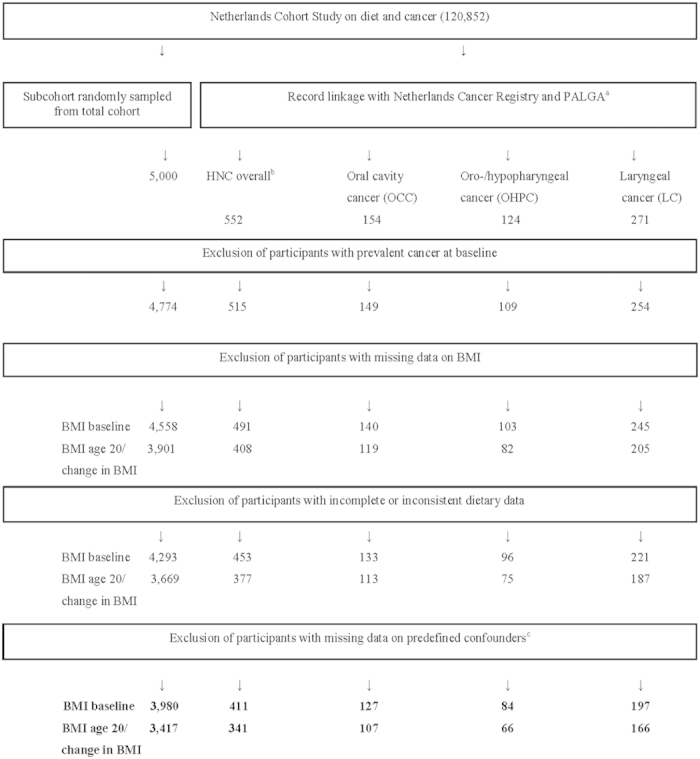
Flow diagram of number of subcohort members and cases on whom the analyses were based. ^a^Abbreviation PALGA: nationwide network and registry of histopathology and cytopathology in the Netherlands. ^b^Oral cavity cancer; oro-/hypopharyngeal cancer; oral cavity, pharynx unspecified or overlapping cancer; laryngeal cancer. ^c^The predefined confounders were age (years), sex, alcohol consumption (grams/day), and cigarette smoking (status (never/former/current), number of cigarettes smoked daily, and number of smoking years).

We excluded cohort members with prevalent cancer other than skin cancer at baseline (Fig. 1). Participants with incomplete/inconsistent dietary data or missing data on confounding variables (see below) were also excluded from analysis^17^,^18^. Only microscopically confirmed first occurrences of squamous cell carcinomas were included^1^,^3^. These comprise nearly all malignancies of the mouth, pharynx, and larynx.

Data for statistical analysis were available for 3,980 subcohort members and 411 incident cases of the selected HNC subtypes (Fig. 1). HNC subtypes were classified as proposed by Hashibe *et al.*^19^, according to the International Classification of Diseases for Oncology (ICD-O-3)^20^ (Table 6). Of the 411 HNC cases, 127 were oral cavity cancer (OCC), 84 were oro-/hypopharyngeal cancer (OHPC), three were oral cavity/pharynx unspecified or overlapping (only included in analyses of HNC overall), and 197 were laryngeal cancer (LC) cases.

### Questionnaire data

At baseline, all participants completed a self-administered questionnaire about habitual dietary intake, lifestyle habits, and other cancer risk factors, including weight, height, and weight at age 20 years. We asked detailed questions about alcohol consumption and cigarette smoking, as described previously^12^. Data were key-entered and processed in a standardized manner, blinded with respect to case/subcohort status in order to minimize observer bias in coding and data interpretation.

BMI at baseline and BMI at age 20 years were calculated using weight at baseline and weight at 20 years, respectively, divided by height at baseline squared (kg/m^2^). We classified BMI at baseline according to the World Health Organization (WHO) standard categories: <18.5 (underweight), 18.5 to <25 (normal weight), 25 to <30 (overweight), and ≥30 kg/m^2^ (obese). For BMI at age 20 years, categories were <20.0, 20.0 to <21.5, 21.5 to <23, 23 to <25, and ≥25 kg/m^2^. We did not use WHO categories here because of few obese cases at the age of 20 years; this classification has been used before in other NLCS analyses^21^. Change in BMI since age 20 years was calculated as BMI at baseline minus BMI at age 20 years and was classified as <0, 0 to <4, 4 to <8, and ≥8 kg/m^2 ^21. Participants with missing values for BMI at baseline were excluded from all analyses; subjects with missing values for BMI at age 20 years were excluded from the analyses of BMI at age 20 years and change in BMI (Fig. 1).

### Statistical analysis

The Cox proportional hazards model was used to estimate age- and sex-adjusted and multivariable-adjusted incidence rate ratios (RR) and corresponding 95% confidence intervals (CI). Person-years at risk were calculated from baseline until diagnosis of HNC, death, emigration, loss to follow-up, or end of follow-up, whichever occurred first. We analyzed BMI at baseline, BMI at age 20 years and change in BMI since age 20 years as described above. For continuous analyses, we used 1 kg/m^2^ as increment in BMI.

To evaluate possible reverse causality, we categorized cases according to the year of follow-up in which they were diagnosed and evaluated BMI of HNC cases during the follow-up period. In addition, we used an independent samples t-test to test for statistical significance of differences between the mean BMI of HNC cases diagnosed during the first two years and cases diagnosed later in follow-up. Based on these results (Table 2), we decided to include the total follow-up time in our analyses. We also performed sensitivity analyses regarding the association between BMI at baseline and risk of HNC overall by excluding the first two years of follow-up.

The predefined confounders were age (years), sex, alcohol consumption (grams/day), and cigarette smoking (status (never/former/current), number of cigarettes smoked daily, and number of smoking years). We considered the following potential confounders: level of education; non-occupational physical activity; energy-intake; consumption of total vegetables, total fruits, fish, red meat, and meat products (all grams/day), and family history of HNC^3^,^22^. None of these variables changed the RR for BMI (continuous) for HNC overall or any of the HNC subtypes by >10% when including them in the model. Therefore, the final model included only the predefined confounders. Analyses of change in BMI were also adjusted for BMI at age 20 years. When adjusting for cigarette smoking frequency and duration, we centered these continuous variables as proposed by Leffondré *et al.*^23^.

We assessed tests for linear dose-response trends by fitting ordinal exposure variables as continuous terms. Standard errors were estimated using the robust Huber-White sandwich estimator to account for additional variance due to sampling from the cohort. The proportional hazards (PH) assumption was assessed using the scaled Schoenfeld residuals^24^. If there was an indication for violation of the assumption for a variable, it was further investigated by adding a time-varying covariate for that variable to the model. We performed analyses for HNC and all HNC subtypes using a time-varying covariate for current smoking, as described before^25^.

To determine whether sex, cigarette smoking, or alcohol consumption possibly modify the association of BMI with risk of HNC overall, we estimated RRs in strata of these exposures. Tests for interaction were performed with BMI on a continuous scale and *P* values for interaction were assessed by including cross-product terms in the models and performing a Wald test. We performed analyses in strata of alcohol consumption (abstainers, consuming >0 to 15 grams ethanol/day, consuming ≥15 grams ethanol/day) and cigarette smoking status (never /former/current). Alcohol consumption and cigarette smoking were mutually adjusted in these models.

All reported *P* values were based on two-sided tests and considered statistically significant if <0.05. Analyses were done using the Stata 13.1 statistical software package (StataCorp, College Station, Texas, USA).

## Additional Information

**How to cite this article**: Maasland, D. H. E. *et al.* Body mass index and risk of subtypes of head-neck cancer: the Netherlands Cohort Study. *Sci. Rep.* **5**, 17744; doi: 10.1038/srep17744 (2015).

## Figures and Tables

**Table 1:**
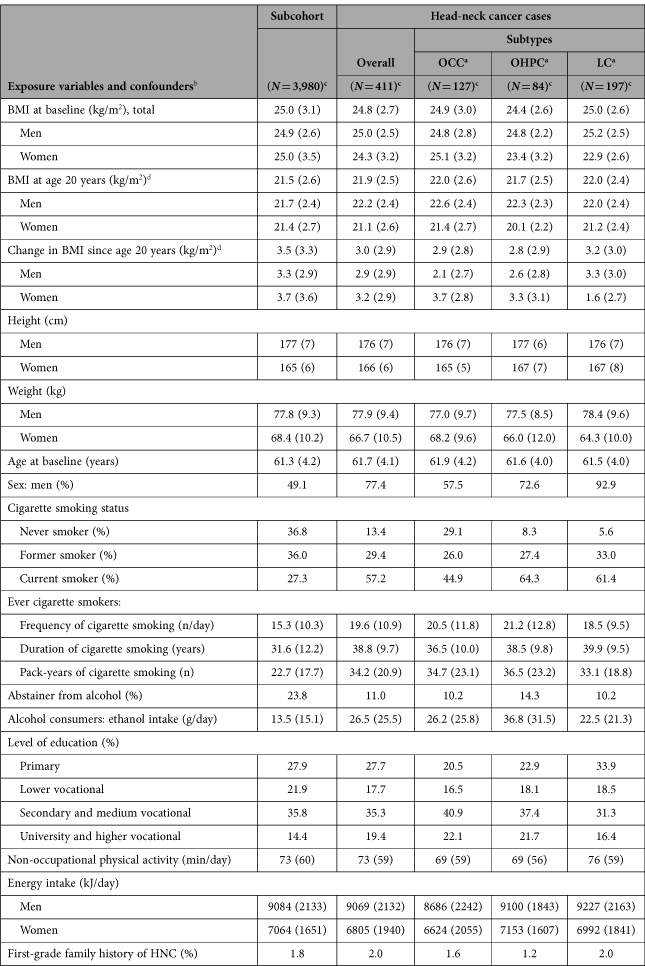
Characteristics of cases and subcohort members; Netherlands Cohort Study, 1986–2006.

^a^OCC: oral cavity cancer; OHPC: oro-/hypopharyngeal cancer; LC: laryngeal cancer.

^b^Values are given as mean (SD); for categorical variables, *N* (%) is presented.

^c^The number of subcohort members or cases (with complete data on BMI at baseline, age, sex, cigarette smoking, and alcohol consumption), used in analyses of BMI at baseline.

^d^The numbers of subcohort members or cases (with complete data on BMI at baseline, BMI at age 20, and change in BMI, age, sex, cigarette smoking, and alcohol consumption), used in analyses of BMI at age 20 and change in BMI: 3,417 subcohort members; 341 HNC overall, 107 OCC, 66 OHPC, and 166 LC cases.

**Table 2:**
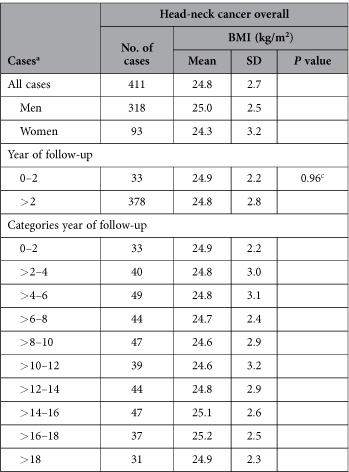
BMI in head-neck cancer (HNC) cases according to sex and time between baseline and HNC diagnosis; Netherlands Cohort Study, 1986–2006.

^a^Mean ± standard deviation (SD) of BMI at baseline in subcohort members were 24.9 ± 2.6 kg/m^2^ for men (*N* = 1,954) and 25.0 ± 3.5 kg/m^2^ for women (*N* = 2,026).

^b^HNC: head-neck cancer; OCC: oral cavity cancer; OHPC: oro-/hypopharyngeal cancer; LC: laryngeal cancer.

^c^*T*-test of mean BMI at baseline in first two years of follow-up vs. rest of follow-up years.

**Table 3:**
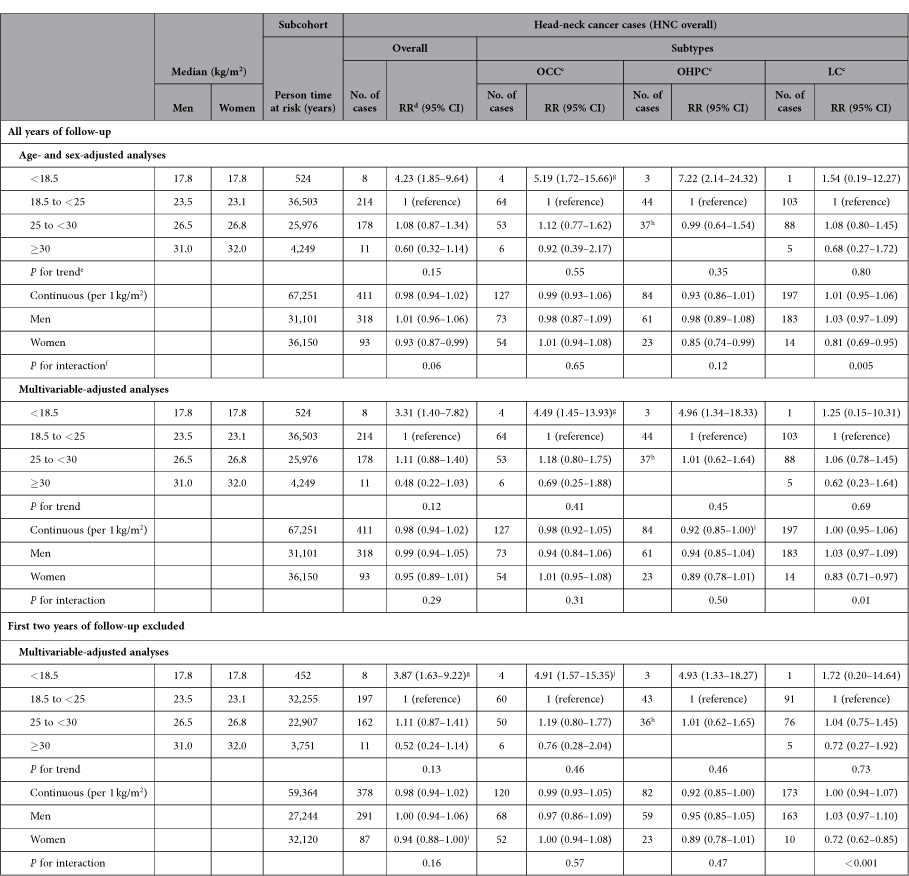
Age- and sex- and multivariable-adjusted^a^ associations between BMI at baseline^b^ and risk of head-neck cancer subtypes; Netherlands Cohort Study, 1986–2006.

^a^Adjusted for age (years), sex, cigarette smoking (status (never/former/current), frequency (number of cigarettes per day; continuous, centered), duration (number of years; continuous, centered)), and alcohol consumption (grams ethanol per day; continuous).

^b^Categories of BMI (kg/m^2^).

^c^OCC: oral cavity cancer; OHPC: oro-/hypopharyngeal cancer; LC: laryngeal cancer.

^d^Abbreviations: RR: incidence rate ratio; CI: confidence interval.

^e^Tests for dose-response trends were assessed by fitting ordinal exposure variables as continuous terms in the Cox proportional hazards model.

^f^*P* Value for interaction between sex and BMI at baseline (continuous), based on cross-product terms in the Cox proportional hazards model and Wald test.

^g^The proportional hazards assumption was possibly violated for the exposure variable in this analysis; there was a statistically significant interaction between the exposure variable and time.

^h^For analyses regarding BMI at baseline and OHPC, BMI was categorized into three categories (<18.5; 18.5 to <25; and ≥25 kg/m^2^) because there were no OHPC cases with a BMI ≥ 30 kg/m^2^.

^i^*P* < 0.05.

^j^The proportional hazards assumption was possibly violated for the exposure variable in this analysis; there was no statistically significant interaction between the exposure variable and time.

**Table 4:**
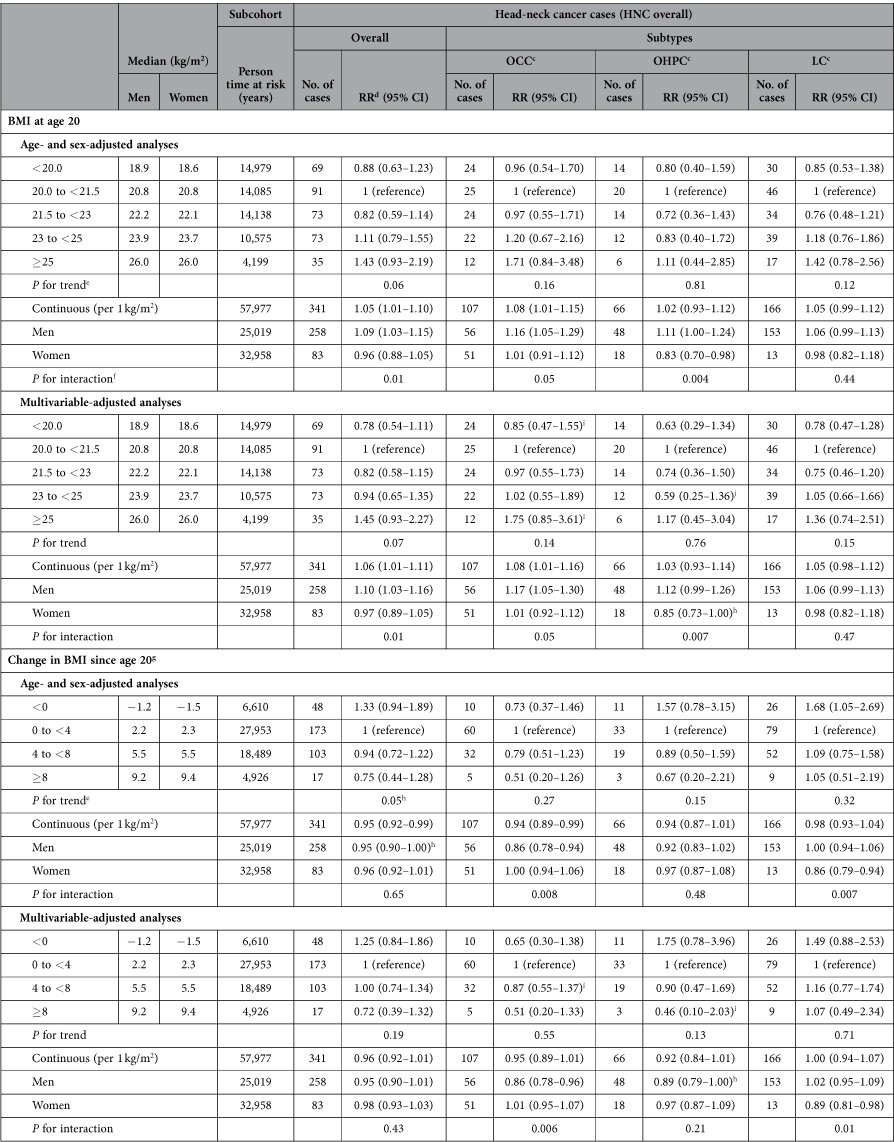
Age- and sex- and multivariable-adjusted^a^ associations between BMI at age 20, change in BMI since age 20^b^ and risk of head-neck cancer subtypes; Netherlands Cohort Study, 1986–2006.

^a^Adjusted for age (years), sex, cigarette smoking (status (never/former/current), frequency (number of cigarettes per day; continuous, centered), duration (number of years; continuous, centered)), and alcohol consumption (grams ethanol per day; continuous).

^b^Categories of BMI at age 20 and change in BMI (kg/m^2^).

^c^OCC: oral cavity cancer; OHPC: oro-/hypopharyngeal cancer; LC: laryngeal cancer.

^d^Abbreviations: RR: incidence rate ratio; CI: confidence interval.

^e^Tests for dose-response trends were assessed by fitting ordinal exposure variables as continuous terms in the Cox proportional hazards model.

^f^*P* Value for interaction between sex and BMI at baseline (continuous), based on cross-product terms in the Cox proportional hazards model and Wald test.

^g^Change in BMI since age 20 years was additionally adjusted for BMI at age 20 years.

^h^*P* < 0.05.

^i^The proportional hazards assumption was possibly violated for the exposure variable in this analysis; there was no statistically significant interaction between the exposure variable and time.

^j^The proportional hazards assumption was possibly violated for the exposure variable in this analysis; there was a statistically significant interaction between the exposure variable and time.

**Table 5:**
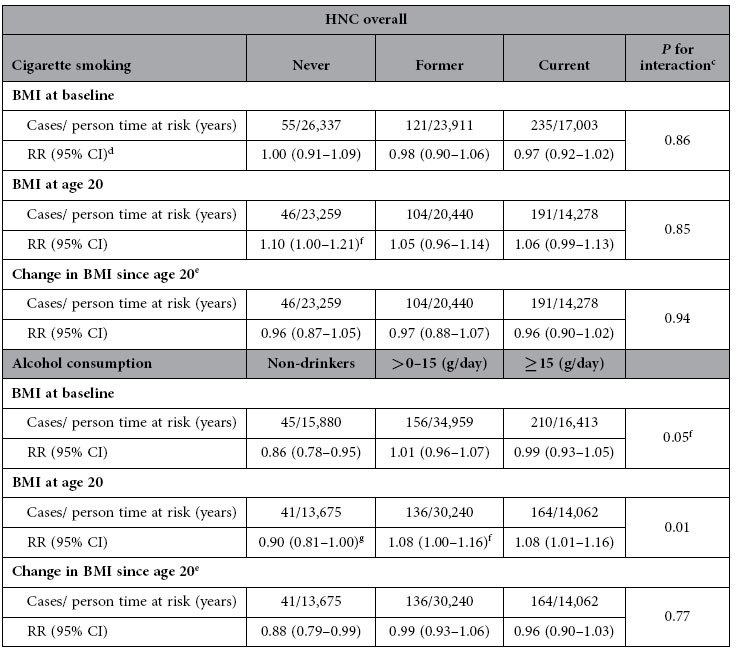
Multivariable adjusted^a^ associations between BMI^b^ and risk of head-neck cancer (HNC) overall, stratified by cigarette smoking status and alcohol consumption; Netherlands Cohort Study, 1986–2006.

^a^Mutually adjusted for age (years), sex, cigarette smoking (status (never/former/current), frequency (number of cigarettes per day; continuous, centered), duration (number of years; continuous, centered)), and alcohol consumption (grams ethanol per day; continuous).

^b^Continuous (per 1 kg/m^2^ increment).

^c^*P* value for interaction based on cross-product terms in the Cox proportional hazards model and Wald test.

^d^Abbreviations: RR: incidence rate ratio; CI: confidence interval.

^e^Change in BMI since age 20 years was additionally adjusted for BMI at age 20 years.

^f^*P* < 0.05.

^g^*P* > 0.05.

**Table 6:** Subclassification of subtypes of head-neck cancer (HNC) as proposed by Hashibe *et al.* 19, according to the International Classification of Diseases for Oncology, version 3 (ICD-O-3)^20^.

HNC-subtype	ICD-O-3
Oral cavity cancer (OCC)	C003-009, C020-C023, C030-C031, C039-C041, C048-C050, C060-C062, C068-C069
Oro-/hypopharyngeal cancer (OHPC)	C019, C024, C051-C052, C090-C091, C098-C104, C108-C109, C129-C132, C138-C139
Oral cavity, pharynx unspecified or overlapping cancer (USC)	C028-C029, C058-C059, C140-C142, C148
Laryngeal cancer (LC)	C320-C329
